# The complete chloroplast genome of *Codonopsis tsinglingensis* (Campanulaceae), an endemic Chinese medicine species in Qinling mountains

**DOI:** 10.1080/23802359.2019.1675543

**Published:** 2019-10-11

**Authors:** Huijuan Zhou, Ruixue She, Peng Zhao, Shuoxin Zhang

**Affiliations:** a College of Forestry, Northwest A&F University, Yangling, China;; b Key Laboratory of Resource Biology and Biotechnology in Western China, Ministry of Education, College of Life Sciences, Northwest University, Xi’an, China

**Keywords:** *Codonopsis tsinglingensis*, complete chloroplast genome, illumina sequencing

## Abstract

*Codonopsis tsinglingensis*, belonging to the Campanulaceae family, is a perennial medicinal herb highly valued in Chinese traditional medicine. The complete chloroplast genome of *C. tsinglingensis* was sequenced using the Illumina Hiseq 4000 platform. The size of the *C. tsinglingensis* chloroplast genome is 170,253 bp, with an average GC content of 38.3%. This circular molecule has a typical quadripartite structure containing a large single copy (LSC) region of 85,408 bp, a small single copy (SSC) region of 8179 bp, and two inverted (IRs) repeat regions of 38,333 bp. The genome contains 138 genes, including 92 protein-coding genes, 38 transfer RNA genes (tRNA), 8 ribosomal RNA genes (rRNA). Phylogenetic analysis based on complete chloroplast genome sequences of 14 species indicates that *C. tsinglingensis*is closely related to *Codonopsis minima* in the Campanulaceae family.

*Codonopsis*is a dicotyledonous genus containing about 42 species of perennial plants,mainly distributed in East, South and Central Asia (He et al. 2015). *Codonopsis tsinglingensis* (Qinling Dangshenin Chinese), is a kind of famous traditional Chinese medicine, which have long been used to lower the blood pressure and treat watery stool with poor appetite (He et al. 2015). Here we have reported first complete chloroplast genome of *C. tsinglingensis* based on Illumina Hiseq 4000 pair-end sequencing data.

The voucher specimen of *C. tsinglingensis* are stored at the herbarium of Northwest University (108°55′E, 34°15′N, accession number: SK2017168). Total genomic DNA was extracted from leaf tissue using the Plant Genomic DNA kit (Tiangen Biotech, Beijing, China). The whole-genome sequencing was conducted with 350 bp pair-end reads on the Illumina Hiseq 4000platform (Illumina, San Diego, CA) by Novogene, Beijing, China. After trimming, the high-quality paired-end reads were assembled with the programme MITObim v1.7 (Hahn et al. 2013) using the *Codonopsis lanceolata* chloroplast genome sequence as a reference (Lee et al. 2018). The chloroplast genome sequence was submitted to GenBank (accession number MN122102).

The chloroplast genome of *C. tsinglingensis*was 170,253 bp in length and contains a pair of inverted repeats (IRa and IRb) regions of 38,333 bp, the large single-copy (LSC) region and small single-copy (SSC) region of 85,408and 8179 bp. A total of 138 genes were successfully annotated containing 92 protein-coding genes, 38 transfer RNA genes, 8ribosomal RNA genes. Among these genes, 12 genes *(atpF*, *ndhA*, *ndhB*, *petB*, *petD*, *rpl2*, *rpl16*, *trnI-GAU*, *trnA-UGC*, *trnK-UUU*, *trnL-UAA*, and *trnV-UAC*) have one intron, and three genes (*rps12, ycf3*, and *clpP*) have two introns.

To conduct phylogenetic analysis, we downloaded complete chloroplast genome sequences of 13 Campanulaceae species from NCBI and *Brassica juncea* as outgroup. The phylogenetic relationships analysis was inferred using the maximum likelihood (ML) method based on complete cp genomes, which was performed using RAxML (Stamatakis 2006). The local bootstrap probability of each branch was calculated by 1000 replications. The resulting tree showed that *C. tsinglingensis* was most closely related to *C. minima* with 100% bootstrap support (Figure 1).

**Figure 1.**
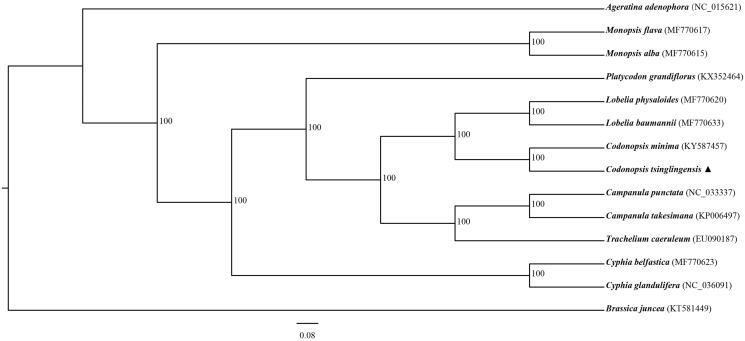
Maximum likelihood (ML) phylogenetic tree based on 14 complete chloroplast genome sequences. The accession numbers showed in the figure, and the triangle indicates that *C. tsinglingensis* in this study.
